# Synthesis and Antimicrobial Activity of New 4-Heteroarylamino Coumarin Derivatives Containing Nitrogen and Sulfur as Heteroatoms

**DOI:** 10.3390/molecules15042246

**Published:** 2010-03-30

**Authors:** Biljana R. Dekić, Niko S. Radulović, Vidoslav S. Dekić, Rastko D. Vukićević, Radosav M. Palić

**Affiliations:** 1 Department of Chemistry, Faculty of Science and Mathematics, University of Priština, Lole Ribara 29, 38220 Kosovska Mitrovica, Serbia; E-Mail: dekic@inbox.com (V.D.); 2 Department of Chemistry, Faculty of Science and Mathematics, University of Niš, Višegradska 33, 18000 Niš, Serbia; E-Mail: radosavpalic@yahoo.com (R.P.); 3 Department of Chemistry, Faculty of Science, University of Kragujevac, R. Domanovića 12, 34000 Kragujevac, Serbia; E-Mail: vuk@kg.ac.yu (R.V.)

**Keywords:** 4-heteroarylaminocoumarins, synthesis, 4-chloro-3-nitrocoumarin, spectral analysis, antimicrobial activity

## Abstract

ynthesis, spectral analysis and bioactivity of new coumarin derivatives are described in this paper. Eight new coumarin derivatives were synthesized in moderate to good yields by condensation of 4-chloro-3-nitrocoumarin and the corresponding heteroarylamine. The synthesized compounds were tested for their *in vitro* antimicrobial activity, in a standard disk diffusion assay, against thirteen strains of bacteria and three fungal strains. They have shown a wide range of activity - from one completely inactive compound to medium active ones.

## 1. Introduction

Coumarin derivatives have been shown to possess a remarkably broad spectrum of biological activity including antibacterial [1,2], antifungal [3,4,5], anticoagulant [6], anti-inflammatory [7], antitumor [8,9] and anti-HIV [10] activity. In addition, these compounds are used as additives in food and cosmetics [11], dispersed fluorescent brightening agents and as dyes for tuning lasers [12]. Main representatives of the class are the hydroxyl derivatives, 4- and 7-hydroxycoumarins, also biologically active and very important for the synthesis of other coumarin derivatives.

On the other hand, the nitrogen and sulfur heterocyclic system families are very interesting due to their physicochemical properties, especially in the sense of design of new drugs and new materials. The chemistry and pharmacology of thiazole derivatives has been of great interest to medicinal chemists lately [13]. The pyrazole ring is a prominent structural moiety found in numerous pharmacologically active compounds. Pyrazole-based derivatives have been regarded as anxiolytics [14], GABA receptor antagonists and insecticides [15], potential PET ligands for CB1 receptors [16], anti-inflammatory, antimicrobial [17], and growth inhibition agents [18].

In continuation of our ongoing interest in synthesis of the new coumarin derivatives [20,21,22,23,24], and having in mind the above considerations, we have been prompted to synthesize new, possibly more potent, pharmacologically active compounds. We decided to combine the coumarinic system with the above named groups of compounds in hope that the resulting novel heterocycles would be biologically active. Additionally, a recent QSAR study of the antimicrobial activity of some 3-nitrocoumarins has put forward some new arguments in this direction [19]. In connection with our previous work [20,21,22,23,24], in the present paper we report on the synthesis of novel 4-heteroarylamino-3-nitrocoumarin derivatives and the screening of their in vitro antimicrobial activity.

## 2. Results and Discussion

**Scheme 1:**
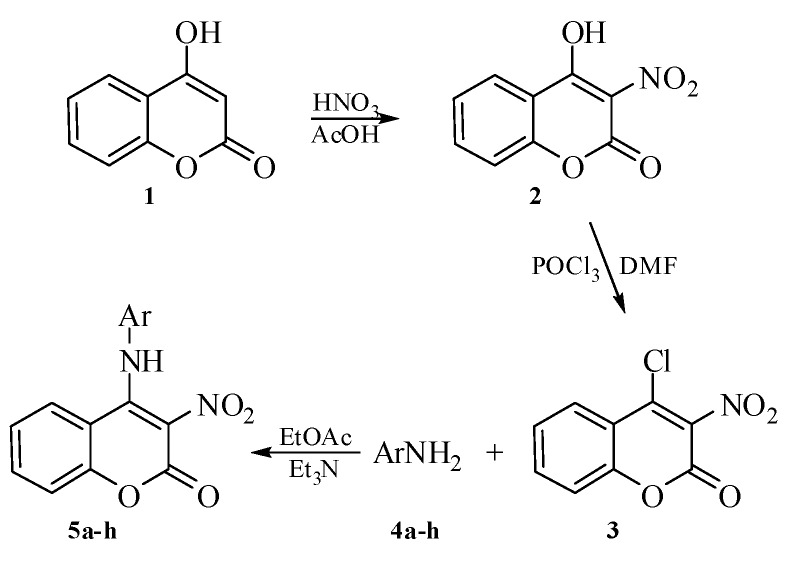
Synthesis of 4-heteroarylamino-3-nitrocoumarin derivatives **5a–h**.

The investigations have been started by synthesis of 4-chloro-3-nitrocoumarin (**3**) applying the known procedure [24], which was, then, submitted to the reaction with the corresponding nucleophiles **4a–h**, all being commercially available [4,5-dihydro-1,3-thiazol-2-amine (**4a**), 5-methyl-1,3-thiazol-2-amine (**4b**), 4-methyl-1,3-thiazol-2-amine (**4c**), 4H-1,2,4-triazol-4-amine (**4d**), 4-amino-1,5-dimethyl-2-phenyl-1,2-dihydro-3H-pyrazol-3-one (**4e**), 5,6-dimethyl-1,3-benzothiazol-2-amine (**4f**), 1H-benz-imidazol-2-amine (**4g**) and 1H-indazol-6-amine (**4h**)]. The reaction has been performed by refluxing the substrate **3**, the corresponding nucleophile **4a–h** and triethylamine in a 1:1:2 ratio (Scheme 1).

The target 4-heteroarylamino-3-nitrocoumarin derivatives **5a–h** were obtained in medium to good yields (66–89%), as it can be seen in Table 1.

**Table 1:** Characterization data of 4-heteroarylamino-3-nitrocoumarin derivatives **5a–h**.

**Compound**	Ar	Formula	Mp/^ o^C	Colour	Yield/ %
**5a**	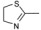	C_12_H_9_N_3_O_4_S	218-220	yellow	75
**5b**	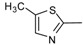	C_13_H_9_N_3_O_4_S	229-232	orange	71
**5c**	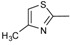	C_13_H_9_N_3_O_4_S	212-215	yellow	72
**5d**	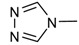	C_11_H_7_N_5_O_4_	249-252	yellow	82
**5e**	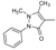	C_20_H_16_N_4_O_5_	240-242	yellow	89
**5f**	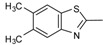	C_18_H_13_N_3_O_4_S	240-242	yellow	66
**5g**	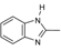	C_16_H_10_N_4_O_4_	253-255	yellow	76
**5h**	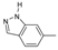	C_16_H_10_N_4_O_4_	255-257	yellow	69

The structures of compounds **5a–h** were confirmed using IR and NMR spectroscopy, and HRMS. The IR spectra of synthesized compounds showed N-H and Ar-H absorptions in the range of 3,040 to 3,391 cm^-1^ and strong bands at 1,656–1,722 cm^-1^ corresponding to absorptions of the C=O and/or C=N bonds. The IR absorptions due to the presence of the 3-NO_2_ group appeared at 1,319–1,382 and 1,523–1,556 cm^-1^. In^ 1^H-NMR spectra, aromatic protons of the coumarin moiety resonated at 7.25–8.45 ppm. The H-6 and H-8 protons were more shielded and appeared as multiplets at 7.25–7.60 ppm. In the case of compound **5g**, H-8 proton signal overlapped with the H-5’ and H-6’ signals of the aryl side group. Protons H-5 and H-7 were more downfield and appeared as doublets of doublets at 8.06–8.45 and doublets of triplets at 7.57–7.95 ppm, respectively. In^ 1^H-NMR spectrum of compound **5h**, thesignal of H-7 overlapped with the resonance of H-4’, while the signal of the proton H-5 in the spectrum of compound **5f** overlapped with the signals of the other aromatic protons of the coumarin moiety and aryl side group of the molecule.

The synthesized compounds **5a–h** were screened for their *in vitro* antimicrobial activity against thirteen strains of bacteria and three fungi strains using a disk diffusion assay [20,25]. The compounds were tested at the dose of 500 μg per disk [50mL of the samples solutions (10 mg/mL) in DMSO]. Measured susceptibility zones to the nearest mm were the clear zones around the disk inhibiting the microbial growth. The obtained results are listed in Table 2. As it can be seen, the prepared compounds possess a wide range of activities - from a completely inactive compound **5e** to medium active ones. The synthesized compounds **5a–h** showed no special selectivity towards any particular microorganism, although the bacterial strains showed greater susceptibility. Also, the activity of the synthesized compounds was slightly higher against Gram-positive compared to Gram-negative bacteria. The largest inhibition zones were noted for *S. aureus* and *S. lutea*. On the other hand, the most resistant strain was *S. cerevisiae*, being almost completely unsusceptible to the tested compounds. Considering the antimicrobial effect towards the different strains of the same microorganism, in the case of *E. coli* ATCC 8739 and *E. coli* ATCC 25922, the latter was less resistant to all compounds. Two different strains of *P. aeruginosa* exhibited similar susceptibility to the tested compounds. The most active compounds were **5c**, **5g** and **5h**, showing reduction of bacterial and fungal growth comparable with the one exhibited by the standards used as positive control (tetracycline and nystatine), especially against medically important pathogens, though in a much larger dose. The lack of susceptibility of all tested microorganisms toward **5e** was probably the consequence of sterically-hindered nitrogen atoms of the pyrazole ring.

**Table 2:** The antimicrobial activity - diameters of growth inhibition zonesof compounds **5a–h** in a disk diffusion assay at a dose of 500 μg per disk.

Microorganism	compound									
	5a	5b	5c	5d	5e	5f	5g	5h	Tetracycline	Nystatine
***B. subtilis***	20	17	20	16	na	16	19	19	27	nt
***Cl. pyogenes***	19	21	21	17	na	20	20	20	27	nt
***Enterococcus*** **sp.**	18	19	22	17	na	20	20	21	28	nt
***M. flavus***	18	19	22	17	na	17	18	20	31	nt
***S. lutea***	20	22	24	18	na	17	23	22	27	nt
***S. aureus***	20	20	27	18	na	19	23	20	25	nt
***E. coli*** ** ATCC 8739**	18	16	20	15	na	18	15	20	27	nt
***E. coli*** ** ATCC 25922**	18	18	24	18	na	20	18	20	28	nt
***K. pneumoniae***	14	12	14	13	na	14	15	na	23	nt
***S. enteritidis***	19	16	22	18	na	20	12	17	26	nt
***P. vulgaris***	20	18	20	17	na	18	18	20	26	nt
***P. aeruginosa*** ** ATCC 27857**	17	19	21	17	na	18	17	21	26	nt
***P. aeruginosa*** ** ATCC 9027**	19	17	22	17	na	19	18	15	25	nt
***A. niger***	12	14	18	14	na	13	15	na	nt	18
***C. albicans***	14	14	15	15	na	14	15	15	nt	19
***S. cerevisiae***	na	na	10	10	na	14	na	na	nt	17

**Figure 1:**
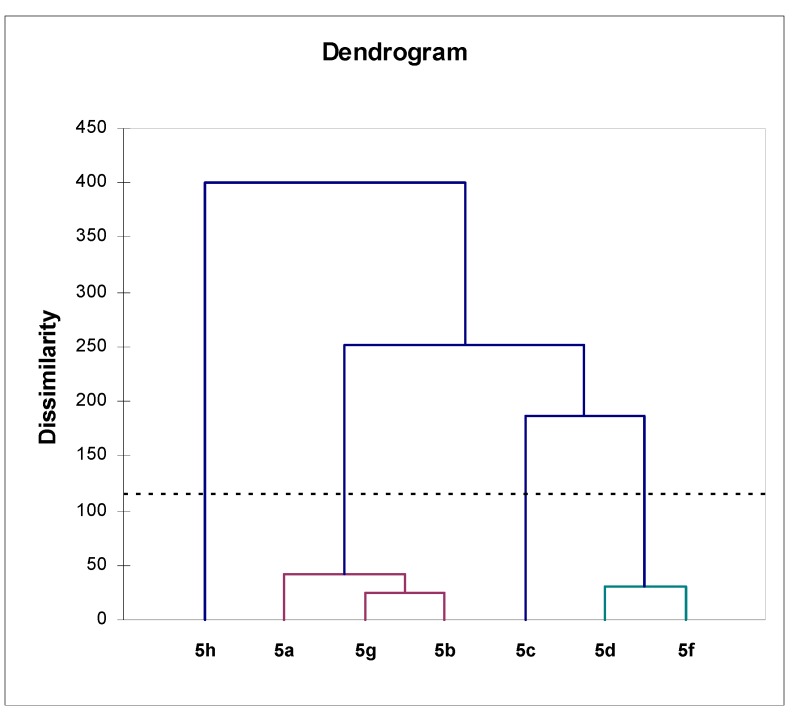
Dendrogram (AHC analysis) representing antimicrobial activity (variables-diameters of growth inhibition zones) dissimilarity relationships of the synthesized compounds (observations) obtained by Euclidian distance dissimilarity (dissimilarity within the interval [0, 400]), using aggregation criterion-Ward’s method. Four groups of the compounds were found.

In order to make the discussion more easy to follow and the conclusions statistically supported, we performed agglomerative hierarchical clustering (AHC) on the mentioned samples (Table 2), using the Excel program plug-in XLSTAT version 2008.6.07. The method was applied utilizing the values of diameters of growth inhibition zones as original variables without any recalculation. The results of AHC are presented in Figure 1. AHC was performed using Pearson dissimilarity (as aggregation criteria simple linkage, unweighted pair-group average and complete linkage were used) and Euclidean distance (aggregation criterion: weighted pair-group average, unweighted pair-group average and Ward’s method). The definition of the groups was based on Pearson correlation, using complete linkage and unweighted pair-group average method. AHC analysis has clearly indicated the existence of four groups of compounds under study (designations of the compounds were given in Scheme 1).

Compound **5e** showed no activity at all towards the tested microorganisms, and was not included in the AHC analysis. A single compound from the first group, **5h**, is distinguished from the rest of the compounds. Structurally speaking, in **5h** the indazole substituent was connected to the coumarin moiety in such a way that the larger distance of the indazole nitrogen atoms from the nitro-group of coumarin moiety might be responsible for the observed activity. No correlation between the structure of the compounds and the observed antimicrobial activity can be drawn out for the compounds placed in the three remaining groups. The marked antimicrobial activity of the synthesized compounds makes the 4-arylamino-3-nitrocoumarin derivatives, with the nitrogen and sulfur as heteroatoms, interesting for further investigation and shows that they are a good basis for the synthesis of new, potentially more physiologically active compounds.

## 3. Experimental Section

### 3.1. General

Melting points were determined on a Kofler hot-plate apparatus and are uncorrected. HRMS(EI) spectra were recorded on a Finnigan-MAT 8230 BE mass spectrometer. The IR measurements (ATR- attenuated total reflectance) were carried out with a Thermo Nicolet model 6700 FTIR instrument. The NMR spectra were recorded on a Varian Gemini 200 spectrometer(^1^H-NMR at 200 MHz,^ 13^C-NMR at 50 MHz), using DMSO-*d*_6_ as the solvent. Chemical shifts are expressed in δ (ppm) using TMS (Me_4_Si) as the internal standard. For TLC, silica gel plates (Kiesel 60 F_254_, Merck) were used. Visualization was affected by spraying the plates with 1:1 aqueous sulfuric acid and then heating. All the reagents and solvents were obtained from commercial sources (Aldrich, USA; Merck, Germany; Fluka, Germany) and used as received, except that the solvents were purified by distillation.

### 3.2. Synthesis of 4-chloro-3-nitrocoumarin ***3***

According to the previously published procedure [26], 4-hydroxycoumarin (**1**) was nitrated in glacial AcOH with 72% HNO_3_ to afford 4-hydroxy-3-nitrocoumarin (**2**). Starting compound **3** was prepared from 4-hydroxy-3-nitrocoumarin (**2**) following the method of Kaljaj *et al*. [27]. The preparation was carried out in the following manner: *N*,*N*-dimethylformamide (DMF, 2 mL, 26 mmol) was cooled to 10 °C in an ice bath. With stirring, POCl_3_ (4 g, 26 mmol) was added dropwise, and the obtained mixture was stirred for an additional 15 min. Then, the ice bath was removed and the reaction was left to proceed at room temperature for a further 15 min. Finally, the solution of 4-hydroxy-3-nitrocoumarin (**2**, 5.4 g; 26 mmol) in DMF (12.5 mL) was added dropwise. After 15 minutes of stirring, the reaction was stopped by adding cold water (15 mL). The precipitated solid was collected by filtration and washed with saturated sodium-bicarbonate solution and water. Recrystallisation from the mixture of benzene-hexane (1:1 volume ratio) yielded yellow crystals of **3** (5.1 g; 22.6 mmol) in 87% yield, mp 162–163 °C. The procedure was repeated twice.

### 3.3. General procedure for the synthesis of 4-heteroarylamino-3-nitrocoumarins ***5a–h***

A solution of 4-chloro-3-nitrocoumarin (**3**, 1 g, 4.4 mmol) and the appropriate heteroarylamine **4a–h** (4.4 mmol) in ethyl acetate (10 mL) was refluxed in the presence of triethylamine (1 mL, 7.2 mmol) for 3–6 h. After cooling, the precipitated solid was filtered off, washed with ethyl acetate and water. The purity of the synthesized compounds was checked by TLC.

*4-(4,5-dihydro-1,3-thiazol-2-ylamino)-3-nitro-2H-chromen-2-one* (**5a**): IR (neat): 3,350 – 3,083 (N-H and Ar-H), 2,945, 1,680 (C=O), 1,605 (C=N), 1,560 (C=C), 1,523 and 1,322 (NO_2_), 1,219, 1,073, 917, 899, 749 cm^−1^;^ 1^H=NMR (DMSO-*d*_6_) δ ppm: 8.29 (*dd*, 1H, H-5, *J* = 1.6, 8.3 Hz), 7.75, (*dt*, 1H, H-7, *J* = 1.4, 8.6 Hz), 7.40–7.54 (*m*, 2H, H-6, H-8), 3.43-3.55 (overlapping signals, 4H; H-4’, H-5’);^ 13^C- NMR (DMSO-*d*_6_) δ ppm: 155.4, 151.1, 147.2, 134.3, 124.8, 124.4, 117.8, 116.5, 114.1, 112.8, 44.5, 32.5; HRMS(EI): M^+^ (C_12_H_9_N_3_O_4_S), 291.2810; requires 291.2826 (Δ = -1.6 mmu).

*4-[(5-methyl-1,3-thiazol-2-yl)amino]-3-nitro-2H-chromen-2-one* (**5b**): IR (neat): 3,367–3,116 (N-H and Ar-H), 2,977 (C-H), 1,682 (C=O), 1,648 (C=N), 1,583 (C=C), 1,523 and 1,382 (NO_2_), 1,204, 1,073, 860, 789 cm^−1^;^ 1^H-NMR (DMSO-*d*_6_) δ ppm: 8.06 (*dd*, 1H, H-5, *J* = 1.4, 8.0 Hz), 7.71 (*dt*, 1H, H-7, *J* = 1.7, 8.4 Hz), 7.34–7.43 (*m*, 2H, H-6, H-8), 7.14 (*brs*, 1H, H-4’), 2.25 (*s*, 3H, CH_3_);^ 13^C-NMR (DMSO-*d*_6_) δ ppm: 155.4, 152.2, 152.1, 134.3 (two C), 126.4, 124.7, 122.7, 122.6, 119.6, 117.8, 117.0, 12.5; HRMS(EI): M^+^ (C_13_H_9_N_3_O_4_S), 303.2951; requires 303.2933 (Δ = +1.8 mmu).

*4-[(4-methyl-1,3-thiazol-2-yl)amino]-3-nitro-2H-chromen-2-one* (**5c**): IR (neat): 3,385–3,175 (N-H and Ar-H), 2,978, 2,945 (C-.H), 1,677 (C=O), 1,599 (C=N), 1,552 (C=C), 1,513 and 1,319 (NO_2_), 1,273, 1,036, 803, 756 cm^−1^;^ 1^H-NMR (DMSO-*d*_6_) δ ppm: 8.06 (*d*, 1H, H-5, *J* = 7.4 Hz), 7.72 (*dt*, 1H, H-7, *J* = 1.7, 8.4 Hz), 7.35–7.44 (*m*, 2H; H-6, H-8), 6.62 (*s*, 1H, H-5’), 2.17 (*s*, 3H, CH_3_);^ 13^C-NMR (DMSO-*d*_6_) δ ppm: 155.5, 155.4, 152.2, 152.1, 134.3, 126.4, 126.3, 124.7, 119.9, 117.8, 117.0, 103.7, 14.0; HRMS(EI): M^+^ (C_13_H_9_N_3_O_4_S) 303.2919; requires 303.2933 (Δ = -1.4 mmu).

*3-nitro-4-(4H-1,2,4-triazol-4-ylamino)-2H-chromen-2-one* (**5d**): IR (neat): 3,391–3,101 (N-H and Ar-H), 2,942, 1,656 (C=O), 1,612 (C=N), 1,595 (C=C), 1,527 and 1,332 (NO_2_), 1,292, 1,034, 929, 798 cm^−1^;^ 1^H-NMR (DMSO-*d*_6_) δ ppm: 8.85 (*s*, 2H, H-3’, H-5’), 8.20 (*dd*, 1H, H-5, *J* = 1.8, 8.1 Hz), 7.62 (*dt*, 1H, H-7, *J* = 1.5, 8.7 Hz), 7.25-7.38 (*m*, 2H*,* H-6, H-8), 2.50 (*s*, 1H, N-H);^ 13^C-NMR (DMSO-*d*_6_) δ ppm: 155.9, 154.2, 151.2, 140.9 (two C), 132.9, 124.8, 124.1, 118.3, 116.8, 112.4; HRMS(EI): M^+^ (C_11_H_7_N_5_O_4_), 273.2051; requires 273.2044 (Δ = +0.7 mmu).

*1,5-dimethyl-4-[(3-nitro-2-oxo-2H-chromen-4-yl)amino]-2-phenyl-1,2-dihydro-3H-pyrazol-3-one*

(**5e**): IR (neat): 3,205 – 3,065 (N-H and Ar-H), 2,920 (C-H), 1,706 (C=O), 1,650 (C=N), 1,611 (C=C), 1,556 and 1,370 (NO_2_), 1,284, 1,056, 895, 794 cm^−1^;^ 1^H-NMR (DMSO-*d*_6_) δ ppm: 9.56 (*s*, 1H, N-H), 8.39 (*d*, 1H, H-5, *J* = 8.0 Hz), 7.79 (*t*, 1H, H-7, *J* = 8.0 Hz), 7.27–7.60 (*m*, 7H, H-6, H-8, H-2”, H-3”, H-4”, H-5”, H-6”), 3.11 (*s*, 3H, CH_3_-N-1’), 2.20 (*s*, 3H, CH_3_-C-5’);^ 13^C-NMR (DMSO-*d*_6_) δ ppm: 161.0, 155.5, 153.1, 151.2, 146.2, 134.8, 134.3, 129.5, 129.4 (two C), 127.3, 125.1, 124.8 (two C), 124.4, 117.7, 113.9, 105.7, 35.5, 10.5; HRMS(EI): M^+^ (C_20_H_16_N_4_O_5_), 392.3662; requires 392.3648 (Δ = +1.4 mmu).

(**5e**): IR (neat): 3,205 – 3,065 (N-H and Ar-H), 2,920 (C-H), 1,706 (C=O), 1,650 (C=N), 1,611 (C=C), 1,556 and 1,370 (NO_2_), 1,284, 1,056, 895, 794 cm^−1^;^ 1^H-NMR (DMSO-*d*_6_) δ ppm: 9.56 (*s*, 1H, N-H), 8.39 (*d*, 1H, H-5, *J* = 8.0 Hz), 7.79 (*t*, 1H, H-7, *J* = 8.0 Hz), 7.27–7.60 (*m*, 7H, H-6, H-8, H-2”, H-3”, H-4”, H-5”, H-6”), 3.11 (*s*, 3H, CH_3_-N-1’), 2.20 (*s*, 3H, CH_3_-C-5’);^ 13^C-NMR (DMSO-*d*_6_) δ ppm: 161.0, 155.5, 153.1, 151.2, 146.2, 134.8, 134.3, 129.5, 129.4 (two C), 127.3, 125.1, 124.8 (two C), 124.4, 117.7, 113.9, 105.7, 35.5, 10.5; HRMS(EI): M^+^ (C_20_H_16_N_4_O_5_), 392.3662; requires 392.3648 (Δ = +1.4 mmu).

*4-[(5,6-dimethyl-1,3-benzothiazol-2-yl)amino]-3-nitro-2H-chromen-2-one* (**5f**): IR (neat): 3,298–3,040 (N-H and Ar-H), 2,935 (C-H), 1,722 (C=O), 1,641 (C=N), 1,600 (C=C), 1,548 and 1,371 (NO_2_), 1,279, 1,061, 869, 755 cm^−1^;^ 1^H-NMR (DMSO-*d*_6_) δ ppm: 7.95 (*dt*, 1H, H-7, *J* = 1.7 Hz, 8.0 Hz), 7.42–7.81 (*m*, 5H, H-5, H-6, H-8, H-4’, H-7’), 7.36 (*s*, 1H, N-H), 2.19 (overlapping signals, 6H, 2CH_3_);^ 13^C- NMR (DMSO-*d*_6_) δ ppm: 154.3, 153.3, 152.8, 152.5, 140.6, 137.0, 136.5, 135.2, 134.1, 126.4, 125.5, 124.0, 119.0, 117.6, 114.1, 113.2, 19.6, 19.3; HRMS(EI): M^+^ (C_18_H_13_N_3_O_4_S) 367.3779, requires 367.3785 (Δ = -0.6 mmu).

*4-(1H-benzimidazol-2-ylamino)-3-nitro-2H-chromen-2-one* (**5g**): IR (neat): 3,125–3,042 (N-H and Ar-H), 2,979, 1,687 (C=O), 1,610 (C=N), 1,573 (C=C), 1,542 and 1,360 (NO_2_), 1,279, 1,075, 804, 756 cm^−1^;^ 1^H-NMR (DMSO-*d*_6_) δ ppm: 8.33 (*dd*, 1H, H-5, *J* = 1.6 Hz, 7.9 Hz), 7.57 (*dt*, 1H, H-7, *J* = 1.7 Hz, 8.2 Hz), 7.37 (*dt*, 1H, H-6, *J* = 1.2 Hz, 8.5 Hz), 7.15-7.29 (*m*, 3H, H-8, H-5’, H-6’), 6.81–7.06 (*m*, 2H, H-4’, H-7’);^ 13^C-NMR (DMSO-*d*_6_) δ ppm: 158.5, 153.0, 150.1, 149.7, 131.4, 129.3, 129.1, 125.7, 124.7, 122.3 (two C), 116.1, 114.8 (two C), 105.7, 99.3; HRMS(EI): M+ (C_16_H_10_N_4_O_4_), 322.2741; requires 322.2750 (Δ = -0.9 mmu).

*4-(1H-indazol-6-ylamino)-3-nitro-2H-chromen-2-one* (**5h**): IR (neat): 3,373–3,075 (N-H and Ar-H), 1,687 (C=O), 1,605 (C=N), 1,590 (C=C), 1,538 and 1,344 (NO_2_), 1,240, 1,107, 876, 759 cm^−1^;^ 1^H- NMR (DMSO-*d*_6_) δ ppm: 13.14 (*brs*, 1H, N-H), 10.42 (*s*, 1H, N-H), 8.45 (*dd*, 1H, H-5, *J* = 1.5 Hz, 7.3 Hz), 8.08 (*s*, 1H, H-3’), 7.70–7.84 (*m*, 2H, H-7, H-4’), 7.44–7.56 (*m*, 2H, H-6, H-8), 7.36 (*d*, 1H, H-7’, *J* = 0.8 Hz), 7.00 (*dd*, 1H, H-5’, *J* = 1.7, 8.5 Hz);^ 13^C-NMR (DMSO-*d*_6_) δ ppm: 155.3, 151.7, 146.1, 139.6, 135.8, 134.5, 133.9, 124.8 (two C), 121.5, 121.2, 117.6, 117.1, 114.9, 114.8, 104.3; HRMS(EI): M+ (C_16_H_10_N_4_O_4_), 322.2739; requires 322.2750 (Δ = -1.1 mmu).

### 3.4. Antimicrobial activity

The *in vitro* antimicrobial activities of compounds **5a–h**were tested against a panel of laboratory control strains belonging to the American Type Culture Collection (Maryland, USA). Antibacterial activity was evaluated against six Gram-positive and seven Gram-negative bacteria. The Gram-positive bacteria used were: *Bacillus subtilis* (ATCC 6633), *Clostridium pyogenes* (ATCC 19404), *Enterococcus* sp. (ATCC 25212), *Micrococcus flavus* (ATCC 10240), *Sarcina lutea* (ATCC 9341) and *Staphylococcus aureus* (ATCC 6538). The Gram-negative bacteria utilized in the assays were: *Klebsiella pneumoniae* (ATCC 10031), *Proteus vulgaris* (ATCC 8427), *Escherichia coli* (ATCC 8739), *Escherichia coli* (ATCC 25922), *Pseudomonas aeruginosa* (ATCC 27857), *Pseudomonas aeruginosa* (ATCC 9027) and *Salmonella enteritidis* (ATCC 13076). The antifungal activity was tested against three organisms *Aspergillus niger* (ATCC 16404), *Candida albicans* (ATCC 10231) and *Saccharomyces cerevisiae* (ATCC 9763).

A disk diffusion method, according to the NCCLS [25], was employed for the determination of antimicrobial activity of compounds **5a–h**. The following nutritive media were used: Antibiotic Medium 1 (Difco Laboratories, Detroit, MI USA) for growing Gram-positive and Gram-negative bacteria, Tripton soy agar (TSA – Torlak, Belgrade) for *C. albicans* and *A. niger*, and Sabouraud dextrose agar (Torlak, Belgrade) for *S. cerevisiae*. Nutritive media were prepared according to the instructions of the manufacturer. All agar plates were prepared in 90 mm Petri dishes with 22 mL of agar, giving a final depth of 4 mm. One-hundred microliters of a suspension of the tested microorganisms (10^8^ cells per mL) were spread on the solid media plates. Sterile filter paper disks (‘‘Antibiotica Test Blattchen”, Schleicher and Schuell, Dassel, Germany, 6 mm in diameter) were impregnated with 50 μL of the samples solutions (10 mg/mL) in DMSO (all solutions were filter-sterilized using a 0.45 μm membrane filter), *i.e.* 500 μg per disk, and placed on inoculated plates. These plates, after standing at 4 °C for 2 h, were incubated at 37 °C for 24 h for bacteria and at 30 °C for 48 h for the fungi. Standard disks of tetracycline and nystatine (origin – Institute of Immunology and Virology ‘‘Torlak”, 30 μg of the active component, diameter 6 mm) were used individually as positive controls, while the disks imbued with 50 μL of pure DMSO were used as a negative control. The diameters of the inhibition zones were measured in millimeters (to the nearest mm) using a ‘‘Fisher-Lilly Antibiotic Zone Reader” (Fisher Scientific Co., USA). Each test was performed in quintuplicate. In order to evaluate statistically any significant differences among mean values, a one-way ANOVA test was used. In all tests the significance level at which we evaluated critical values differences was 5%.

## 4. Conclusions

In summary, this paper describes the synthesis, spectral characterization and screening of antimicrobial activity of some new 4-arylamino-3-nitrocoumarin derivatives. 4-Chloro-3-nitro-coumarin proved to be a good electrophilic substrate in these reactions. The synthesized compounds showed a wide range of potentially promising antimicrobial activities. The notable antimicrobial effect of certain compounds confirms that these are a good basis for the production of a number of new, possibly physiologically active coumarin derivatives.
